# Novel ACE inhibitory tripeptides from ovotransferrin using bioinformatics and peptidomics approaches

**DOI:** 10.1038/s41598-019-53964-y

**Published:** 2019-11-22

**Authors:** Zhipeng Yu, Yang Chen, Wenzhu Zhao, Fuping Zheng, Long Ding, Jingbo Liu

**Affiliations:** 1 Beijing Advanced Innovation Center for Food Nutrition and Human Health, Beijing Technology and Business University (BTBU), Beijing, 102488 P.R. China; 2 College of Food Science and Engineering, Bohai University, Jinzhou, 121013 P.R. China; 3 Lab of Nutrition and Functional Food, Jilin University, Changchun, 130062 P.R. China

**Keywords:** Renovascular hypertension, Renovascular hypertension, Orthopaedics, Orthopaedics

## Abstract

Food-derived ACE inhibitory peptides have recently attracted increased attention. This work focused on a more efficient *in silico* method to find ACE inhibitory peptides from ovotransferrin. In this work, ovotransferrin was digested into peptides by virtual enzymolysis. Subsequently, *in vitro* ACE inhibitory activity of potential tripeptides was conducted following the peptide score, toxicity, and water solubility prediction. Both pharmacophore study and flexible docking were applied to analyze ACE inhibition mechanism of tripeptides. Our results demonstrated that EWL was a potent ACE inhibitory tripeptide with IC_50_ value of 380 ± 10 μM. Besides, pharmacophore and flexible docking showed that the pi interaction and hydrogen bond were the key interactions in ACE-EWL complex. It appears that the *in vitro* ACE inhibitory activity of tripeptide EWL was consistent with its molecular modeling.

## Introduction

Egg white is considered as a rich source of high quality proteins, which can release bioactive peptides after enzymatic hydrolysis^1^. Previous work has demonstrated that egg-derived peptides exerted antioxidant, antimicrobial, and angiotensin converting enzyme (ACE) inhibitory activities^2,3^. Particularly, ACE inhibitory peptides attracted more attention, which can block the active site of ACE and suppression angiotensin-I translate to angiotensin-II, therefore reduce blood pressure^4^. Regarded as an efficient strategy of anti-hypertension (AHT), ACE inhibitors have been studied for years. However, present clinical ACE inhibitors were almost obtained by chemical synthesis. Those synthetic ACE inhibitors were potent and widely used as an anti-hypertension drug, despite their adverse side effects including coughing, allergic reactions, taste disturbances and skin rashes^5^. Therefore, more attentions have been focused on finding less side effects and economically available substitute, like food-derived ACE inhibitory peptides^6^.

In recent years, more and more ACE inhibitory peptides have been found in food proteins^7–9^. However, conventional workflow of ACE inhibitory peptides discovering has several drawbacks, such as time and cost consuming^10^. Indeed, increasing attention has come to the idea of inventing a more efficient method to discover ACE inhibitory peptides from food proteins. As bioinformatics approaches emerged, bioinformatics e-tools and database were integrated to create an efficient and time saving method to discover ACE inhibitory peptides. In fact, some ACE inhibitory peptides have already been successfully discovered using *in silico* analysis^11^. Database information can be easily accessed, *i*.*e*., BIOPEP, NCBI, and UniProt databases. Subsequently, the potential peptides were analyzed using online services ExPASy and Bioware, aimed to predict properties of novel peptides. In particular, molecular docking can help to understand how molecules working together, and predict certain bio-activities.

This study was to discover novel ACE inhibitory peptides from ovotransferrin using *in silic*o approaches. Ovotransferrin from egg white was hydrolyzed *via* virtual digestion. Generated dipeptides and tripeptides were screened by peptide score, toxicity, and water solubility prediction. Subsequently, *in vitro* activity of peptides with the potential to inhibit ACE was assayed. Finally, the molecular mechanisms of potent ACE inhibitory tripeptides were investigated.

## Results

### Protein selected for digital proteolysis

As shown in Table 1, proteins coded in F1NVN3 and Q92062 have the highest sum of four favorable amino acids (Pro, Tyr, Trp, and Phe). Further BLAST analysis result shows that F1NVN3 and Q92062 only have 7 different amino acids out of 738 amino acids. Those differences appeared in amino acid number 73, 304, 332, 350, 422, 591, 716. As described previously, amino acid residues with bulky side chains as well as hydrophobic side chains were preferred for dipeptides to bind with ACE. For tripeptides, the most favorable residues for the carboxyl terminus were aromatic amino acid, positively charged amino acids were preferred for middle position, and hydrophobic amino acids were preferred for the amino acid terminus. According to information above, Protein sequence identity by UniProt as F1NVN3 was chosen to process in virtual proteolysis, and digested into various lengths of peptides.

**Table 1:** Amino acid composition of ten ovotransferrin.

Protein ID	Pro	Tyr	Trp	Phe	Sum
A0A1D5NZF8	25	28	11	29	93
A0A1D5P4L7	28	21	10	27	86
E1BQC2	29	22	10	29	90
E1BUL8	23	25	11	31	90
F1NVN3	26	28	11	30	95
P02789	28	21	10	27	86
Q4ADG4	28	21	10	27	86
Q4ADJ6	28	21	10	27	86
Q4ADJ7	28	21	10	27	86
Q92062	26	28	11	30	95

Numbers in this table are representing quantity of corresponding amino acid.

### Promising peptides selected by certain properties

In the current work, only di- and tripeptides were chosen to have selection with specific properties owing to they can be easily absorbed into the blood circulatory system from the digestive tract. However, the potency of peptides is not only depending on their human digestive tract absorption but also their bioactivity. And toxicity and water solubility were also important properties for peptide employment^12^. As so, three properties include high peptide score, non-toxin, and good water solubility. Peptide score was a score function given by PeptideRanker that predicts peptides bioactivity. In the results, a total of 1276 di- and tripeptides was produced by all 19 enzymes and 1 enzyme combination. All peptides with peptide score higher than 0.6 were predicted to be non-toxin, but peptides with good water solubility seem pretty limited. Various sequenced peptides may lead to more different potent ACE inhibitory peptides, Refer to the results, pancreat elastase and stem bromelain can produce most different kind and most quantity of qualified peptides, stem bromelain have 6 different kind of promising peptides; while pancreat elastase have 5 different kind of promising peptides. Sequence EWL and CDL had occurred in both digest result. All those peptides (*i*.*e*., CDL, CR, EF, EWL, FRS, KDF, KMF, RL, and RWI) have not been published as ACE inhibitory peptides and applied to later molecular docking project.

### ACE inhibitory activity prediction by molecular docking

As shown in Table 2. All nine eligible peptides have -CDOCKER ENERGY values higher than lisinopril has, however only KDF have higher -CDOCKER INTERACTION ENERGY values than lisinopril. For -CDOCKER ENERGY, Both EWL and KDF have much higher score than all the others, For -CDOCKER INTERACTION ENERGY value, there are more high scored peptides, such as EWL, KDF, KMF and RWI. When put both scoring values in considering, EWL and KDF were chose to have chemical synthesis. Even though, KMF and RWI have higher -CDOCKER INTERACTION ENERGY values than EWL, but still seems in the same level. While EWL have much higher -CDOCKER ENERGY value than KMF and RWI, it is the highest -CDOCKER ENERGY scored peptide. -CDOCKER ENERGY and -CDOCKER INTERACTION ENERGY were the scoring function of CDOCKER algorithm. -CDOCKER ENERGY represented the ligand strain energy and receptor-ligand interaction energy. And -CDOCKER INTERACTION ENERGY was non-bonded interaction that exists between the protein and the ligand^13,14^.

**Table 2:** Virtual docking simulation of eligible peptides.

Peptide	-CDOCKER ENERGY (kcal/mol)	-CDOCKER INTERCTION ENERGY (kcal/mol)
CDL	87.2882	71.1951
CR	84.4289	70.1488
EF	94.1159	81.0789
EWL	106.4860	90.7678
FRS	92.8453	81.6175
KDF	103.8760	101.9490
KMF	94.0595	92.9380
RL	88.0132	87.4242
RWI	84.4705	92.6442
Lisinopril	77.0264	95.1048

### *In vitro* ACE inhibitory activity test of chose peptides

**Figure 1:**
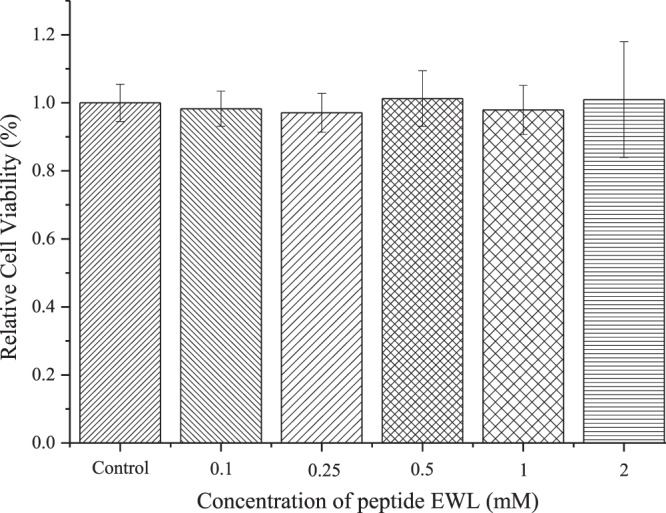
Effect of EWL on HepG_2_ cells. Cells were treated with EWL at the indicated concentrations (0.1, 0.25, 0.5, 1 and 2 mM), cell viability was assessed by the MTT assay. *p < 0.05 compared with the control group.

ACE inhibitory activity of chosen promising peptides, *i*.*e*., KDF and EWL, were performed by High Performance Liquid Chromatography (HPLC) method. It turned out that EWL can de-active ACE efficiently with IC_50_ value of 380 ± 10 μM. However, KDF can de-active ACE’s catalytic reaction for about 14.27% in concentration of 0.45 mM. After another two confirming test at concentration of 0.45 mM. We finally decided not to find out KDFs’ IC_50_ value owing to it’s extremely low suppressant activity. Moreover, the cytotoxicity of EWL was examined using MTT toward HepG2, results indicated that the high concentration EWL was low-toxicity (shown in Fig. 1). At room temperature, EWL can be dissolved in water and is soluble up to 4 mM in water, the solubility of EWL in water was greater than 1.78 g/L.

### Interaction mechanism of ACE-tripeptides

**Figure 2:**
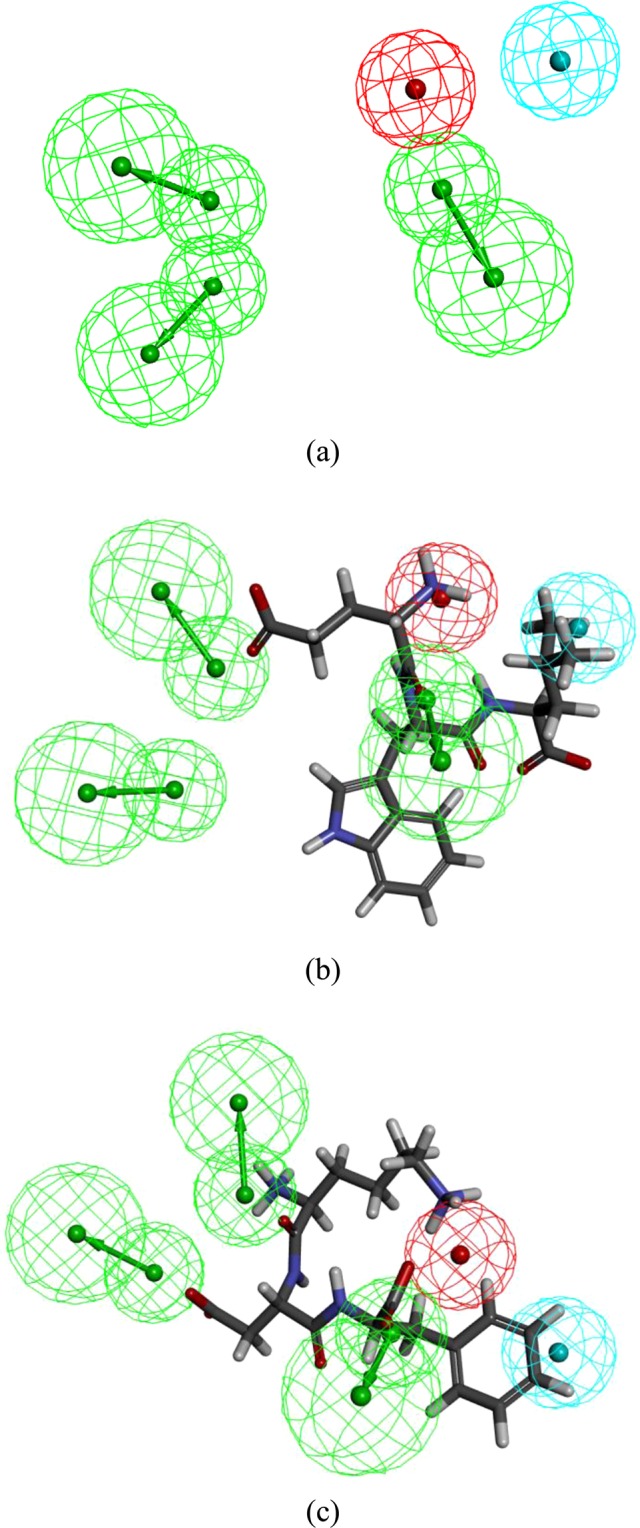
Elite pharmacophore model generated based on EWL and KDF: (**a**) Pharmacophore-3 model. (**b**) Mapping of the tripeptide EWL to pharmacophore-3. (**c**) Mapping of the tripeptide KDF to pharmacophore-1. (Purple ball stand for HBD feature, blue ball stand for negative charge, azure ball stand for hydrophobic feature, red ball stand for positive charge).

**Figure 3:**
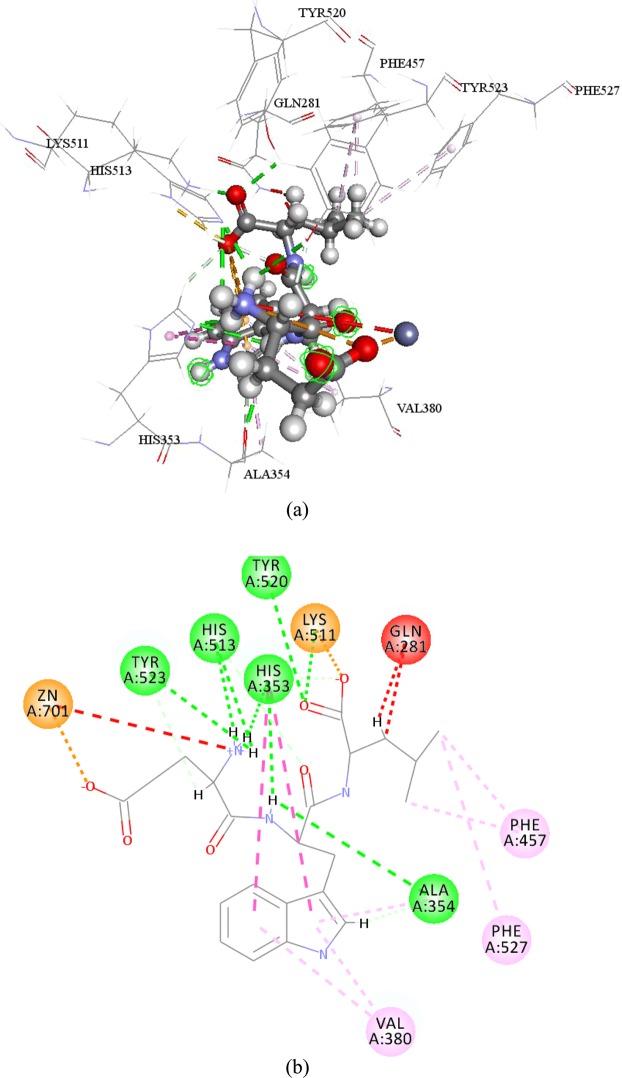
3D (**a**) and 2D (**b**) diagram of interactions of EWL-ACE complex. Green dash line represent conventional hydrogen bond, brown dash line represent attractive charge, violet dash line represent pi interaction, indigo dash line represent carbon hydrogen bond, and red dash line represent unfavorable charge.

**Figure 4:**
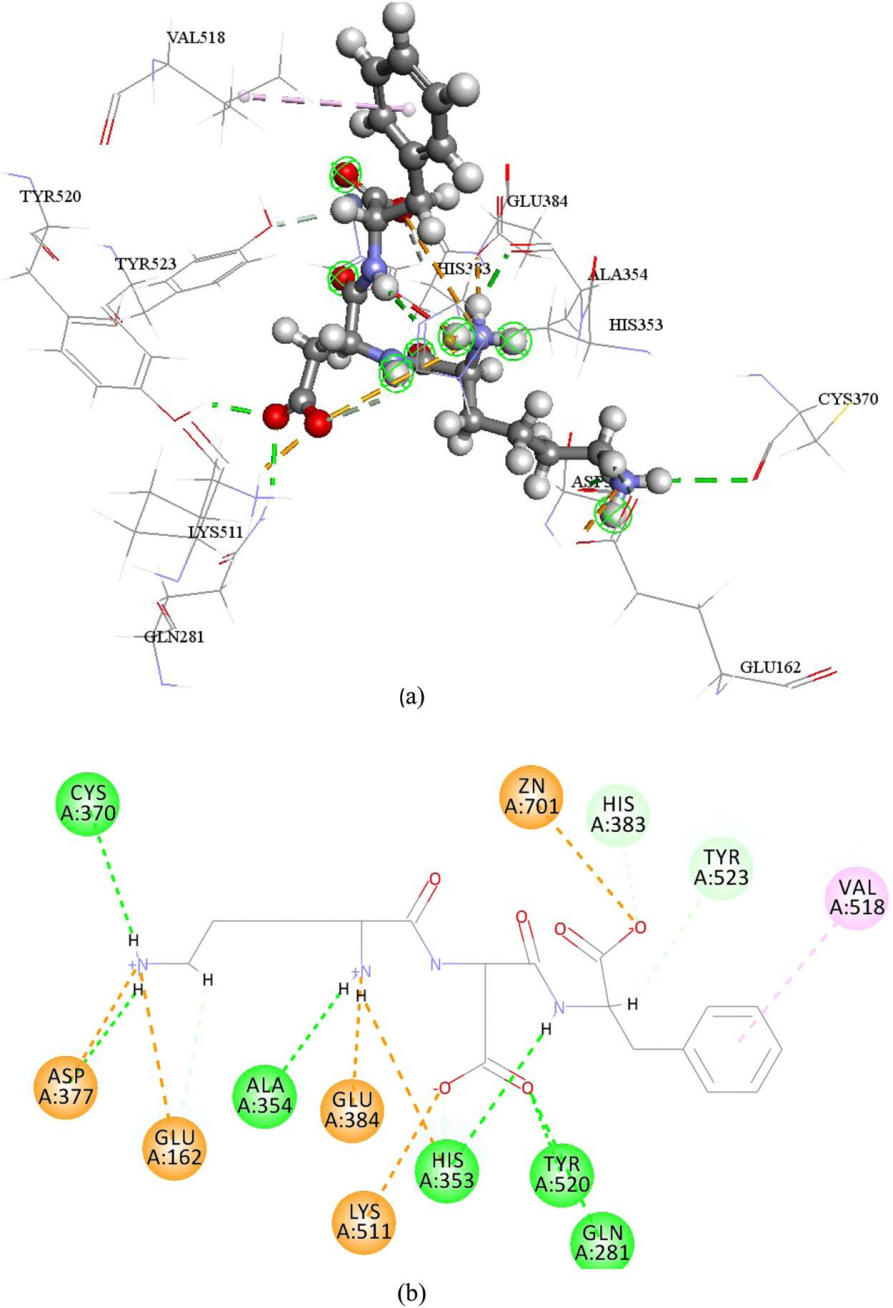
3D (**a**) and 2D (**b**) diagram of interactions of KDF-ACE complex. Green dash line represent conventional hydrogen bond, brown dash line represent attractive charge, violet dash line represent pi interaction, indigo dash line represent carbon hydrogen bond.

**Figure 5:**
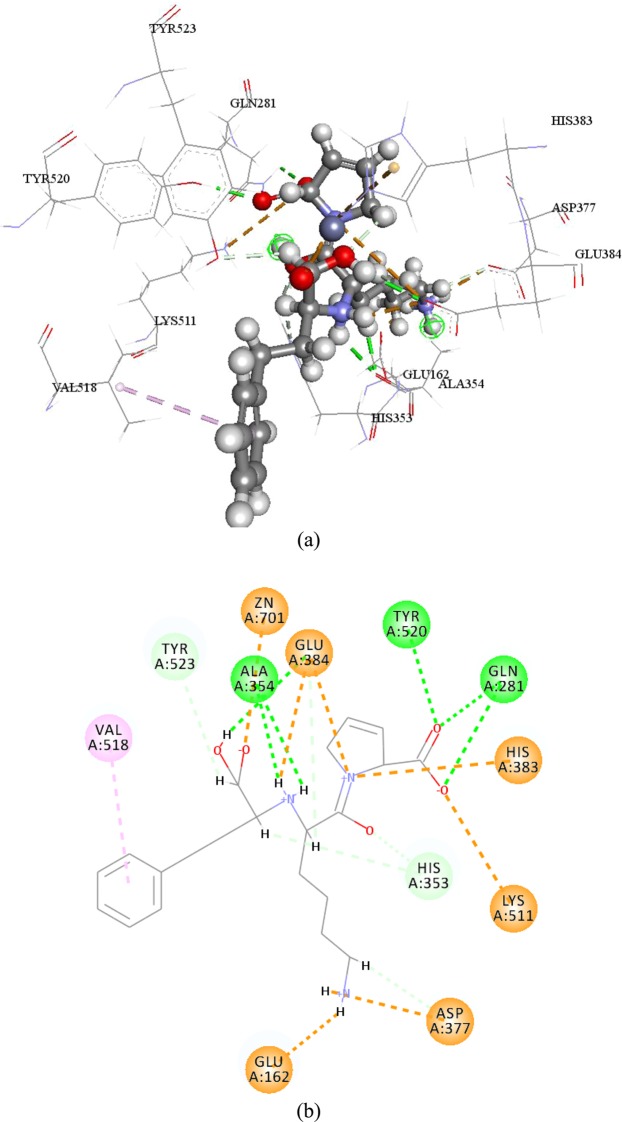
Ligand interaction of lisinopril-ACE complex in 3D (**a**) and 2D (**b**) diagram. Green dash line represents conventional hydrogen bond, brown dash line represents attractive charge, violet dash line represents pi interaction, and indigo dash line represents carbon hydrogen bond.

Pharmacophore research was finished hoping to find particular ACE binding preferred features in ligand level. This research will calculate a fit value for the pharmacophore model align to two different peptides, which reveal the fit degree between pharmacophore model and each peptide. In previous study, HipHop program of Discovery Studio 2017 was used to generate 10 pharmacophore models. And pharmacophore-3 consisted of three hydrogen bond acceptors (HBA), one hydrophobic region (Hyd), and one positive ionizable (PI) was the best model (shown in Fig. 2a)^15^. The mapping results showed that pharmacophore-3 estimated the high fit value of 3.23045 for the active peptide EWL and low fit value 0.25080 for the peptide KDF. The mapping on the tripeptide EWL (shown in Fig. 2b) showed that HBA3 located on the oxygen atom O17, and one PI located on the nitrogen atom N1 of the amine group. But, the tripeptide KDF (shown in Fig. 2c) could not match thoes features well. To detail the binding affinity and interface interactions between ACE and inhibitory peptides, Flexible docking was utilized. According to induce fit theory, substrate working with enzyme requires conformation changes of both enzyme and substrate. But it would cost enormous money and time to construct and scoring conformations of whole protein structure. Flexible Docking can produce conformations of acceptors’ active site. Although Flexible docking only generate conformations of residues in accepter active site, it can still improve accuracy of docking result^16^. Flexible docking results of EWL and KDF were shown in Figs 3 and 4. Crystal structure of angiotensin converting enzyme, 1o86 was the first revealed ACE C-domain structure^17^, and it could better predict the ACE bioactive and was widely used in various ACE docking studies^18^. To compare the docking result with known drug lisinopril, here present interactions of lisinopril-ACE complex in Fig. 5. The flexible docking showed that both EWL and KDF can dock into ACE S1 and S2’ pocket, while KDF can docked into one more S1’ pocket^19^.

## Discussion

There are 10 ovotransferrin sequences from egg white in UniProt database. After calculating amino acid composition with ProtParam service, protein with highest frequency of four favorable amino acids (Pro, Tyr, Trp and Phe) was chosen to conduct further research. Even though, proline was well documented as the most favorable amino acid for peptides binding to ACE^20^, the selection of four favorable amino acids was not made by putting frequency of proline in the first priority. However, the selected protein sequence was chose by the simply summary of all favorable amino acid frequency, aim to find various constitute ACE inhibitory peptides; multiple constituted peptides can lead to a comparison in later mechanism study. And peptides can possess high ACE inhibitory activity without comprise proline.

When properties of bioactive peptides limited in high peptide score, non-toxin, good water solubility and unpublished ACE inhibitory peptide, only one potent ACE inhibitory peptide showed up. However, after digestion by stem bromelain and pancreat elastase there are 7 published ACE inhibitory peptides and they also confirmed as peptide score over 0.6 and predict to be non-toxin. Those 7 peptides are MF, WL, WA, IF, MG, YG and FG. Stem bromelain digestion result comprise one MF; WL; IF; MG; KDF; EWL and two WA and YG. Pancreat elastase results in one WL; FG; MG EWL and two WA. It seems, digestion of egg white ovotransferrin by stem bromelain can lead to maximum non-toxin high scored peptides. Digestion of ovotransferrin by human gastrointestinal protease was also been investigated. Two proving ACE inhibitory peptides have been found in the results, GR and GF^21^. This indicate that ovotransferrin have theoretic ACE inhibitory after human digestion tract, but still too soon to say that egg can regard as an anti-hypertension strategy food. For human digestion of food include digestion by not only protease release from human body but also human gut microbiota, and digestions by human gut microbiota were not quite revealed. Hence, the yield of the two peptides after gastrointestinal digestion would still in myth.

To the number of all kinds of interaction, there are 8 conventional hydrogen bonds, 2 carbon hydrogen bond, 8 pi interaction, 2 attractive charge, and 3 unfavorable interactions in EWL-ACE complex; 6 conventional hydrogen bond, 2 carbon hydrogen bond, 1 pi interaction, and 5 attractive charge in KDF-ACE complex. Due to EWL’s potent ACE inhibitory activity, this result indicates that pi interaction also play an important role in peptide binding with ACE. There are previous paper had written about the importance of hydrogen bond in ligand complexes binding with ACE^22^. In this study, the mapping results showed peptide EWL could match HBA and PI features better than KDF, and the hydrogen bonds in EWL were more concentrated than those in KDF.

In flexible docking results, even though, KDF can dock into three active pocket of ACE, it still cast low ACE inhibitory activity. It seems how many pocket ACE inhibitory peptides can bind with ACE isn’t ACE inhibitory activity determinant element. Except residues determined in the three active pocket, (Ala354, Glu384, Tyr523 in s1; Gln281, Tyr520, Lys511, His513 and His353 in S2’ and Glu162 in S1’), the residues of ACE bind with EWL and KDF were different. Both lisinopril and EWL have complex binding results. It seems some residues would like to interact with different atoms at a certain distance. This may give ACE ability to immobilize peptides more stabilized which may lead to higher inhibitory ability.

Interactions with zinc ion was always been mention for it playing key role in forming metal-acceptor interaction^23^. EWL can interact with ACE zinc atom by an attractive charge. Although KDF can form interaction with zinc ion but possess low ACE de-active activity. It seems interactions with zinc atom were not very important in inhibit ACE in this research, which against previous study^24^. Zinc ion has tetra-coordinate with three ACE residues (His383, His387, Glu411). And the distortion of the tetra-herald geometry can cause ACE inhibitory activity^25^. However, this phenomenon was not detected in this study. In other words, the bond length between zinc ion and the three mentioned residues were not change, in other word, the bond length remain in 2.07086 Å with His387, 1.99577 Å with Glu411 and 2.03772 Å with His383. It seems that even zinc ion have well documented as an important factor for peptide interact with ACE, peptides ACE inhibitory activity can not been determined only by interaction with zinc ion.

## Materials and Methods

### Selection of protein

Ovotransferrin make up about 12% of egg white protein content, which can be extracted in large quantities and is available to apply in food industry. Ovotransferrin was specially chosen in this study for it is the main ingredients in egg white and its reasonable sequence length^26^. Different ovotransferrin sequences from egg white were published in UniProt database. As bioactivity of peptides are owing to their amino acid composition. Di- and tripeptides consisted of particular amino acids (Pro, Tyr, Trp, and Phe) was in favor of binding with ACE^27^. In the present work, those amino acids were applied to find the sole sequence with most possibility of producing potent ACE inhibitory peptides among the ovotransferrin sequences collected from UniProt database. Composition of all those sequences was analyzed in ProtParam, available at https://web.expasy.org/protparam/. A further amino acid sequence comparison was made by BLAST, available at https://blast.ncbi.nlm.nih.gov/Blast.cgi.

### *In silico* proteolysis of ovotransferrin

Variety in proteases could lead to enormous differences in the bioactivity of peptides. Ovotransferrin was digested by different enzymes, aim to produce more di- and tripeptides for further screening, as well as select the enzyme which could produce maximum number of ACE inhibitory peptides. Total 19 enzymes and 1 enzyme combination (simulating of gastrointestinal proteolysis) were utilized to accomplish this digital proteolysis. Enzymes were gathering for their using in food industry^28^. This subject was completed by using webservers PeptideCutter (available at https://web.expasy.org/peptide_cutter/) and BIOPEP analysis ENZTME(S) ACTION (available at http://www.uwm.edu.pl/biochemia/index.php/en/biopep)^28^. Di- and tripeptides were chosen to have specific properties elimination for they can be absorbed directly into the blood circulatory system from the digestive tract^29^. Nineteen enzymes, *i*.*e*., cathepsin G, chymase, chymosin, clostripain, ficin, glutamyl endopeptidase II, leukocyte elastase, oligopeptidase B, oligopeptidase F, pancreat elastase, pancreat elastase II, papain, pepsin (pH = 1.3), pepsin (pH < 2), proteinase K, proteinase P1, stem bromelain, thermolysin, trypsin and V8 (pH 7.8) were utilized to digest ovotransferrin.

### Physiochemical properties and toxic prediction of potential tripeptides

Some physiochemical properties were very important for bioactive peptides exhibiting biological activity in human body^30^. And toxicity would be another vital nature for drugs and bioactive peptides. In this part, bioactivity, toxin and solubility were employed to be the key parameters for choosing the qualified peptides to have ACE inhibitory activity prediction (molecular docking with ACE crystal structure 1o86). Bioactivity prediction was made by Peptide Ranker (available at http://bioware.ucd.ie/compass/biowareweb/), and toxicity tests were finished by ToxinPred (available at http://crdd.osdd.net/raghava/toxinpred/multi_submit.php). PepCalc (available at http://pepcalc.com/) was utilized to conduct water solubility prediction. Di- and tripeptides with peptide ranker score over 0.6, non-toxicity and good water solubility prediction were put into continued subjects. Meanwhile, chosen peptides were not identified in previous study from BIOPEP database.

### Prediction of ACE inhibitory activity of tripeptides by molecular docking

It has been demonstrate that ACE inhibitors can competitively and unselectively block the active sites of ACE, therefore deactivate the course of angiotensin-II^31^. This means if peptides can bind with ACE tight, it could have ACE inhibitory activity. The ACE crystal structure (PDB ID: 1o86) was downloading from RCSB PDB, which bonded with lisinopril. The docking site was chosen from the five build-in active site in crystal structure, active site AC5, for it have docked lisinopril in its center and including zinc ion. However the radius of final docking active site was set at 9 Å. ACE crystal structure would been through two steps before docking started. Firstly, add hydrogen. Secondly, delete water and irrelevant atoms. Discovery Studio 2017 R2 was used to draw out the 3D structure of promising peptides as well as docking them to ACE crystal structure (1o86). Structures of these peptides were optimized by Dynamics simulation and prepare ligands protocol before docking into ACE. Docking protocol was calculated using CDOCKER algorithm, it would give out the values of CDOCKER ENERGY and CDOCKER INTERCTION ENERGY. The docking was carried out with coordinates x: 40.6183, y: 34.4676, and z: 44.7269 with a radius of 9 Å. The pose cluster radius were set to 0.1 Å. The random conformations were set to 10, the orientations to refine was set to 10, and the simulated annealing was set to true. Peptides with lower -CDOCKER ENERGY and -CDOCKER INTERACTION ENERGY values were selected for *in vitro* ACE inhibitory activity assay.

### Promising peptides synthesis

Potential peptides were synthesized by the solid phase procedure peptide using FMOC protected amino acids synthesis methods with an AAPPTEC 396 Automated Peptide Synthesizer (Advanced Automated Peptide Protein technologies, USA). Detials have been written in our previous work^32^.

### *In vitro* ACE inhibitory activity assay

*In vitro* ACE inhibitory activity was measured followed by high-performance liquid chromatography 15 with some modification. More details seen in our previouslystudy^7^.

### Cytotoxicity verification of EWL

To verify the accuracy of the toxicity prediction results, we used a cell assay to detect the cytotoxicity of EWL. After the HepG 2 cells were seeded and pre-incubated, the cells were treated with 10 μL EWL (0.1, 0.25, 0.5, 1 and 2 mM final concentration). Cell viability in this experiment was determined by the MTT [3-(4,5-dimethylthiazol -2-yl)-2,5-diphenyltetrazolium bromide] assay and absorption Value was measured at 490 nm.

### Ligand based pharmacophore study

Ligand phamacophore mapping of Discovery Studio 2017 R2 Client was used to fits the tripeptides to the phamacophore generated in previous study. The conformation generation was set to fast, and the maximum omitted features was set at -1. All other parameters were left in their default configurations.

### Flexible docking of ACE-tripeptides

Ligand based pharmacophore study can help to understand ACE inhibitory mechanism in ligand level. Flexible docking, a more accurate docking protocol than CDOCKER, was employed to dock KDF and EWL into ACE crystal structure (1o86). Flexible docking is the protocol that allows the key residues in acceptor become movable; further lead to position changing of atoms in both ligand and acceptor to meet the best conformation. Key movable residues in ACE were selected as the residues in three ACE active sites: S1 (Ala354 Glu384 Tyr 523), S1’ (*i*.*e*., Gln281,Tyr520, Lys511, His513, and His 353) and S2’ (Glu162)^33^.
